# Supplementary material for: Endophytic Fungal Diversity of Mangrove Ferns Acrostichum speciosum and A. aureum in China
Source: Plants (Basel). 2024 Feb 29;13(5):685. doi: 10.3390/plants13050685 (PMC10935002; doi:10.3390/plants13050685)
Supplement: Supplementary file 1 [file plants-13-00685-s001.zip › Table S1.pdf]

Table S1. Results of ITS sequencing for *Acrostichum speciosum* and *A. Aureum* in Hainan, Wenchang, and Zhanjiang are presented. The first letter H, W, and Z represent Haikou, Wenchang, and Zhanjiang, respectively. The second letter J and L represent *A. speciosum* and *A. aureum*, respectively. The third letter L, P, R, and RS represent leaf, petiole, root, and rhizome, respectively. The numbers 1, 2, and 3 represent the three biological replicates of each sample.

SampleID	Raw_total_reads	Clean_total_reads	Clean_total_tags	Q20(%)	Q30(%)	GC(%)
H-J-L1	91181	90764	87301	100	99.7	59.2
H-J-L2	92104	91934	88777	100	99.7	58.9
H-J-L3	88257	88135	85307	100	99.7	59.6
H-J-P1	84928	84405	79738	99.9	99.7	56.6
H-J-P2	90448	90337	87517	100	99.7	57.4
H-J-P3	89916	89722	85594	99.9	99.7	58.6
H-J-R1	61762	61319	54391	99.9	99.4	44.3
H-J-R2	84683	83244	37186	99.9	99.5	37.7
H-J-R3	49807	49656	45229	99.9	99.1	39.8
H-J-RS1	85142	84998	81674	100	99.7	45.8
H-J-RS2	90699	89357	63876	99.9	99.4	48.3
H-J-RS3	90275	87994	69830	99.9	99.2	50.2
H-L-L1	89223	85136	72905	99.9	99.5	47
H-L-L2	86548	84957	75534	99.9	99.4	50.8
H-L-L3	88679	88349	80789	99.9	99.5	46.2
H-L-P1	35626	35527	33453	99.8	99	51.7
H-L-P2	76441	75785	62429	99.9	99.3	50.2
H-L-P3	86305	86177	82757	99.8	99	53.1
H-L-R1	87624	86239	76744	99.9	99.5	50
H-LR2	88903	88804	85692	100	99.7	50.1
H-L-R3	90523	90158	84264	99.9	99.5	53.4
H-L-RS1	89055	88910	85375	99.9	99.7	51.4
H-L-RS2	87124	86864	82104	100	99.7	49.8
H-L-RS3	90339	89982	85044	100	99.8	48.9
W-J-L1	88163	88053	85413	99.9	99.7	57.4
W-J-L2	85408	84514	80257	99.9	99.7	58.8
W-J-L3	87095	86898	83726	99.9	99.7	58
W-J-P1	91369	90108	85026	99.9	99.6	56.8
W-J-P2	87951	87500	81687	99.9	99.6	56.3
W-J-P3	91078	90763	85805	99.9	99.7	56.2
W-J-R1	87854	87492	82356	99.9	99.2	40.5
W-J-R2	88111	87868	82347	99.9	99.2	40.7
W-J-R3	90635	90292	83868	99.9	99.3	41.9

W-J-RS1	68994	68615	62644	99.9	99.6	46.3
W-J-RS2	88512	88156	82507	99.9	99.6	44.9
W-J-RS3	86939	86732	83249	99.9	99.6	46.8
W-L-L1	37369	37141	34154	99.9	99.5	52.5
W-L-L2	86574	86359	79954	99.9	99.5	51.1
W-L-L3	85065	84894	78410	99.9	99.3	53.8
W-L-P1	88104	79960	58820	99.9	99.4	46.7
W-L-P2	86658	79528	63109	99.9	99.3	47.2
W-L-P3	87433	87299	81579	99.9	99.5	46.8
W-L-R1	88594	88485	85108	100	99.8	49.7
W-L-R2	90507	90101	85478	99.9	99.4	49.9
W-L-R3	88581	88364	84583	100	99.7	48.9
W-L-RS1	88020	87851	84296	100	99.8	47.7
W-L-RS2	87085	85821	73925	99.9	99.5	48.6
W-L-RS3	85463	85349	83017	99.9	99.7	51.5
Z-J-L1	88880	84456	71263	99.9	99.5	45.7
Z-J-L2	85268	84913	76784	99.9	99.6	45.5
Z-J-L3	88691	88319	82371	99.9	99.4	47.1
Z-J-P1	90825	90615	80927	99.8	99.3	50.5
Z-J-P2	91720	91277	82764	99.9	99.6	48.5
Z-J-P3	91686	90963	82940	99.9	99.6	45.5
Z-J-R1	88311	88150	82036	100	99.7	47.1
Z-J-R2	89300	88821	76973	100	99.7	45.4
Z-J-R3	88863	88413	77345	99.7	98.8	49.1
Z-J-RS1	85165	84989	80211	99.9	99.5	52.1
Z-J-RS2	86923	86693	82622	100	99.7	49.2
Z-J-RS3	89434	89319	85019	100	99.7	49.5
Z-L-L1	89361	88902	79662	99.9	99.7	44.6
Z-L-L2	77182	76404	69082	99.9	99.5	50.9
Z-L-L3	91763	91155	84218	99.9	99.5	46.5
Z-L-P1	89864	89108	70754	99.9	99.5	49.4
Z-L-P2	87639	86742	69348	99.9	99.6	48.4
Z-L-P3	84269	83640	77122	99.9	99.5	45.3
Z-L-R1	88523	88382	84540	100	99.8	46.6
Z-L-R2	90461	90375	86809	99.8	98.9	48
Z-L-R3	90661	89823	83055	99.9	99.6	44.5
Z-L-RS1	85867	85804	82804	99.8	99	48.2
Z-L-RS2	92165	92007	88069	100	99.7	46.9
Z-L-RS3	86007	85849	83578	99.8	98.9	47.6
