# Supplementary material for: Endophytic Fungal Diversity of Mangrove Ferns Acrostichum speciosum and A. aureum in China
Source: Plants (Basel). 2024 Feb 29;13(5):685. doi: 10.3390/plants13050685 (PMC10935002; doi:10.3390/plants13050685)
Supplement: Supplementary file 1 [file plants-13-00685-s001.zip › Table S3.pdf]

Table S3. Unique endophytic fungi in different species, different locations, and different tissues. For the unique endophytic fungi of *Acrostichum speciosum* or *A. aureum*, the superscript letters Z, W, and H respectively indicate that the unique endophytic fungi are only distributed in one location (Zhanjiang, Wenchang, or Haikou), and the letters L, P, R, and RS respectively indicate that the unique endophytic fungi are only distributed in one tissue (leaves, petiole, roots, or rhizomes), and if there is no superscript letter, it means that the unique endophytic fungi are distributed in two or more locations or tissues. The unique endophytic fungi of a certain location or tissue exist simultaneously in *A. speciosum* and *A. aureum*.

***Acrostichum speciosum* Willd. only**	***Acrostichum aureum* L. only**	**Zhanjiang area only**	**Wenchang area only**	**Haikou area only**	**Leaf only**	**Petiole only**	**Root only**	**Root stock only**
Alfaria [H,R]	Achroiostachys	Coprinellus	Scleroramularia	Verruculina	/	Umbelopsis	/	Pileomyces
Alloconiothyrium [Z,RS]	Acrophialophora	Waitea	Phialemonium	Eupenidiella				Deconica
Apiosordaria [Z,RS]	Anthracocystis [Z,R]	Gliocladiopsis						Lasionectria
Apodus [H,L]	Arthrographis	Septobasidium						Graphium
Beltrania [L]	Ascobolus	Lodderomyces						Exidia
Cabalodontia [W,P]	Ascotaiwania [H,RS]	Kockovaella						Trematosphaeria
Calonectria [Z.R]	Badarisama	Mariannaea						
Conidiobolus [Z,L]	Bartalinia [Z,R]	Golubevia						
Cordana [W,RS]	Basidiobolus	Saitozyma						
Curvibasidium [Z,L]	Bionectria [Z]	Paraglomus						
Cystobasidiopsis [Z,L]	Bisifusarium [Z,RS]	Setophoma						
Debaryomyces [W,RS]	Blakeslea [Z,RS]	Pseudopestalotiopsis						
Endophragmiella [L]	Blastobotrys	Montagnula						
Epulorhiza [H,RS]	Brachyphoris [Z,RS]	Pileomyces						

Geminibasidium [R]
Hemileucoglossum [W]
Hermatomyces
Heterochaete
Knufia [Z,P]
Kwoniella [W,P]
Leptosphaerulina [W,RS]
Lunulospora [W,R]
Marasmius [Z,P]
Metacordyceps [W]
Microcera [Z,RS]
Monodictys [W,P]
Myriangium [Z,L]
Myxocephala
Myxospora [Z,RS]
Panaeolus [Z,L]
Phlebiopsis
Pseudofusicoccum
Pyrenula [Z,RS]
Readerielliopsis [Z,RS]
Rhodosporidiobolus
Ruinenia [RS]
Sistotremastrum [W,P]
Stomiopeltis [Z,L]
Sympodiomycopsis [Z,L]
Tremella

Bryochiton [Z,RS]
Chaetosphaeronema
Chalara [L]
Choanephora
Chrysosporium [H,RS]
Ciboria
Claroideoglomus
Clinoconidium [Z,L]
Coprinopsis
Cortinarius [Z]
Cyberlindnera [Z,RS]
Daldinia
Dinomyces [Z,RS]
Discosia [Z,R]
Diversispora [Z,R]
Duddingtonia
Entoloma
Funneliformis
Gamsia
Geastrum [Z,RS]
Gibellulopsis [Z,RS]
Golovinomyces [Z,L]
Grammothele
Halosarpheia
Hansfordia [Z,L]
Hemimycena [Z,R]

Lentinus
Graphium
Gigaspora
Clavulina

Volutella [Z,RS]
Wickerhamomyces [Z,RS]

Hirsutella [Z,L]
Hormonema [W,P]
Hyalorbilia [Z,RS]
Iodophanus [W,P]
Issatchenkia
Itersonilia [Z,RS]
Lactarius [W,P]
Latorua [W,P]
Lectera [Z,RS]
Lentinula [Z,RS]
Leotia [Z,L]
Macrophomina
Magnaporthe [Z,RS]
Metapochonia [RS]
Minimedusa
Monosporascus [Z,RS]
Mucor [Z]
Mycoarthris [Z,R]
Mycotribulus [Z]
Myrothecium
Neoascochyta
Ochroconis [Z]
Paecilomyces [Z]
Paraphoma
Peniophorella [H,L]
Peziza

Phanerochaete [Z,RS]
Phyllozyma
Poaceascoma [Z,RS]
Polystigma
Pseudaleuria
Pseudopithomyces
Pseudozyma [Z]
Ramariopsis [Z,R]
Ramicandelaber [Z,RS]
Recurvomyces [Z,RS]
Rhexodenticula [Z]
Rhizophlyctis
Rhizophydium
Russula [Z]
Sagenomella
Savoryella [H]
Sclerotinia
Scolecobasidium [Z,R]
Setophaeosphaeria [H,RS]
Sonoraphlyctis [Z,R]
Sordaria
Sphaeropsis
Spissiomyces
Stagonospora [Z,RS]
Striatibotrys [Z,R]

Tetracladium
Thielavia [Z,R]
Trametes [H,P]
Trechispora [RS]
Tricharina [Z,R]
Westerdykella
Wiesneriomyces [Z,R]
Xylaria [Z,RS]
